# Economic burden of vertigo: a systematic review

**DOI:** 10.1186/s13561-019-0258-2

**Published:** 2019-12-27

**Authors:** Eva Kovacs, Xiaoting Wang, Eva Grill

**Affiliations:** 1 Institute for Medical Information Processing, Biometrics and Epidemiology, Ludwig-Maximilians-Universität München, Munich, Germany; 2 German Center for Vertigo and Balance Disorders, Faculty of Medicine, University Hospital, Ludwig-Maximilians-Universität München, Marchioninistr. 15, 81377 Munich, Germany; 3 Munich Center of Health Sciences, Ludwig-Maximilians-Universität München, Marchioninistr. 15, 81377 Munich, Germany

**Keywords:** Vestibular vertigo, Dizziness, Cost of illness, Health care costs cost analysis, Health care utilization

## Abstract

**Background:**

Vertigo, a highly prevalent disease, imposes a rising burden on the health care system, exacerbated by the ageing of the population; and further, contributes to a wide span of indirect burden due to reduced capacity to work or need of assistance in activities of daily living. The aim of this review was to summarise the evidence on the economic burden of vertigo.

**Methods:**

All original studies published between 2008 and 2018 about the economic evaluation of peripheral or central vestibular vertigo in developed countries were considered eligible, unrestricted to setting, health care provider, or study type.

**Results:**

The electronic search in three databases identified 154 studies from which 16 qualified for inclusion. All studies presented partial economic evaluation referring to a variety of vestibular vertigo including unspecified vertigo. Nine studies presented monetised cost results and seven studies reported health care utilization. Direct costs derived mainly from repeated and not well-targeted consultations at all levels of health care, excessive use of diagnostic imaging, and/or of emergency care. Considerable societal burden was caused by decreased productivity, mainly due to work absenteeism.

**Conclusion:**

To the best of our knowledge, this is the first systematic review of the existing evidence of the economic burden of vertigo. The lack of conclusive evidence revealed apparent targets of future research. First, studies of diagnostics and therapies for vestibular disease should include cost-effectiveness considerations. Population-based studies of health services utilization should include simple vestibular assessments to get more reliable estimates of the burden of disease and associated costs on the level of the general population. Further, clinical and population-based registries that include patients with vestibular disease, should consider collecting long-term data of societal burden. Primary data collection should increasingly include assessment of health care utilization e.g. by linking their diagnoses and outcomes to routine data from health insurances.

## Background

Vertigo and dizziness, belonging to the most frequent symptoms with an estimated lifetime prevalence of 17–30% [1], cover diseases and conditions of various origin [2, 3]. In a narrower sense, vertigo refers to peripheral or central vestibular diseases with a lifetime prevalence of up to 10% [1, 4] and a yearly incidence of 1.4% [4]. The most prevalent types of peripheral vestibular vertigo are benign paroxysmal positional vertigo (BPPV), Meniere’s disease (MD), vestibular neuritis and bilateral vestibulopathy; vestibular migraine is one of the most common examples of central vestibular vertigo [3, 5].

Arguably, vertigo and dizziness are among the main drivers for health care utilization from primary care [6–8] through specialist care [9] to tertiary level hospitals [10]; therefore may have a high impact on direct costs in industrialized countries. The increasing prevalence of vertigo in older population [11] further contributes to this burden of health care [12].

Individuals with acute symptoms may present at all levels of the health system including emergency services [13, 14]; however most instances of vertigo might be diagnosed and treated at the primary care level [15]. This is often not the case, leading to unjustified diagnostic procedures, prolonged time to diagnosis and repeated specialist consultations [6]. One major driver of direct health care costs may be the overutilization of imaging procedures, which actually would have a well-defined but limited role in differentiating vestibular disease from rare but life-threatening conditions such as stroke [16]. Regarding therapy in BPPV, the most prevalent type of vertigo in older adults [4], liberatory manoeuvres [17, 18] may bring fast relief [19]. Pharmacotherapy has its place in a limited number of pathologies [20]; vestibular rehabilitation [21] should be offered to all patients with vestibular deficiency [22].

Regarding indirect costs, vertigo can be a reason for sick leave and occupational disability. In a study from the United Kingdom (UK) and Italy, patients reported a mean of 7 days absence from work due to dizziness in the previous 6 months [23]. From those patients still working, over 50% felt that their work efficiency had dropped [23]; over one fourth of them had changed their jobs and 21% had quit work [23]. Likewise, in Belgium, more than half of the patients of a tertiary dizziness center reported having been on sick leave due to dizziness, and 12% were completely unable to return to work [24].

Also, vestibular disease may cause considerable restrictions of activities of daily living [23] and quality of life [25]. Loss of quality of life from vestibular disease was recently estimated to amount to a total of 64,929 USD lifetime economic burden per patient, or in a total lifetime societal burden of 227 billion USD for the USA population over 60 years of age [26].

As financial burden of disease seems to be considerable for vertigo and dizziness, we aimed to summarise the evidence from available quantitative studies in a systematic way. Specifically, we aimed to summarize information on costs arising from diagnosis, referral or therapy.

## Methods

In this review the Preferred Reporting Items for Systematic Reviews and Meta-Analyses [27] was followed.

### Search strategy and eligibility criteria

All original studies regarding any kind of economic evaluation of vertigo were considered irrespective of study design or perspective (e.g. payer or society).

The detailed search strategy for the electronic databases Medline, EMBASE, and Cochrane Library is presented in the Additional file 1 Beside the general terms “vertigo” or “dizziness”, the search terms were compiled to cover the above listed most prevalent vertigo types of peripherial or central vestibular origin, and functional vertigo; including both their referring Medical Subject Headings (MeSH) terms and additional free text. Restricting the search to title and abstract ensured to focus on studies handling vertigo or dizziness as the main topic of the study. Vestibular Schwannoma was excluded with respect to its surgical treatment being not comparable with the conservative management of all other vertigo types. MeSH terms representing a broad range of economic evaluation were completed with a free text search.

With respect to the comparability of economic results, developed countries according to the categorization of the United Nations Statistics Division [28] were selected. The hits were restricted to being published in English in the last 10 years, i.e. between October 2008–01. October 2018.

Full texts were retrieved via the online library service of the Ludwig-Maximilians-Universität München. We contacted two authors for further information unsuccessfully. The search in the electronic databases was not extended by a handpicked search. Study selection was performed by two independent researchers (XW and EK). Disagreement was resolved by discussion or in lack of consensus by the decision of a third researcher (EG).

### Data collection

A Microsoft Excel form was prepared (XW) for collecting data of study characteristics, vertigo type(s), and outcome indicators. Assessing methodological quality and risk of bias, the list of Consensus on Health Economic Criteria (CHEC) [29] was applied. One researcher (XW) performed the data extraction and a second researcher (EK) checked the data.

### Conversion of the economic results

Costs expressed in national currencies and in different price years were reported both in original form and converted into 2016 USD using The Campbell and Cochrane Economics Methods Group Evidence for Policy and Practice Information Coordinating Centre (CCEMG –EPPI-Centre) Cost Converter [30]. The converter adjusts the price year according to the Gross Domestic Product deflator index; while conversion between countries/currencies is based on Purchasing Power Parities, in accordance with the recommendation of the Cochrane Handbook for Systematic Reviews [31].

In case of studies reporting health care utilization in units as an outcome, health care utilization in units as an outcome, despite considerable efforts we were not able to retrieve historical unit prices for converting reported units into monetary estimates.

## Results

### Study selection

**Fig. 1:**
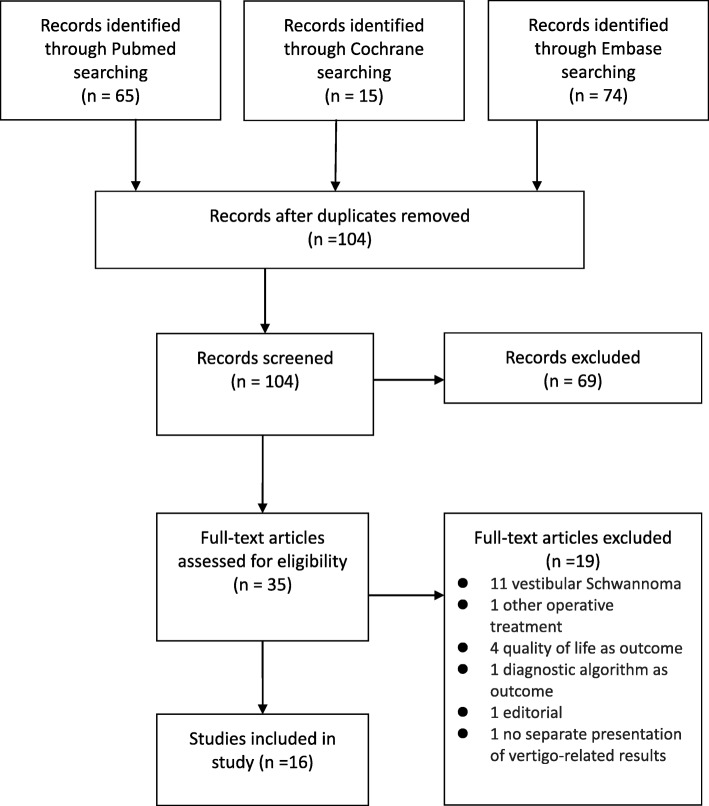
Flowchart of study selection

The electronic search identified 154 studies from three databases. After removing duplicates, 104 studies were screened by title and abstract according to the eligibility criteria. Inclusion was discussed in 21 cases, resulting in 35 studies qualifying for full-text reading. Disagreement was then solved by discussion in 17 cases, and in lack of consensus in six cases, the third researcher made the decision. Sixteen studies were included in the review. Figure 1 presents the flow of study selection.

### Study characteristics

Detailed description of the original studies, the setting, timeframe, included vertigo type(s), the selection criteria, the main characteristics of the study population, and the reported type of burden was presented in Table 1. The 16 studies covered seven countries: the USA (seven studies [32–34, 36, 38, 42, 44];), the UK (three studies [41, 45, 47];), Germany (three studies [37, 40, 46];), Canada (one study [39];), Norway (one study [43];) and a multicentre study [35] from which we selected the data referring to the developed countries in Europe, namely the Czech Republic, Germany, Hungary, and Slovenia. Health care providers were primary care [47], emergency department in four studies [33, 34, 39, 42], and hospital in three studies [36, 41, 44]. Two studies covered more than one sector of the health care system [35, 37]; and five studies applied a population-based approach [32, 38, 40, 45, 46].

**Table 1:** Characteristics of selected studies

Study, country	Study design, data source	Setting	Time frame	Included vertigo type	Study sample (size, selection criteria, age, gender)	Type of reported burden
Adams, M.E., et al., 2017 [32]; USA	retrospective review of Medicare data Health Care Financing Administration Common ProcedureCoding System	different geographic regions of the USA	2000–2010	dizziness, not otherwise specified, vertigo of central origin; MD; BPPV; other peripheral vestibular disease	5798 patients with vestibular testing from 63,578 with vestibular diagnosis; from total 231,984 patients • inclusion: age ≥ 65 • mean age not reported; 68% female	resource use for diagnostic investigations in rate of population
Ahsan, S.F., et al., 2013 [33]; USA	retrospective chart review	emergency department	January 2008–January 2011	ICD–9 codes 386.11 (BPPV) or 780.4 (Dizziness and giddiness)	1681 patients • inclusion: ED visit due to vertigo; being assigned to the health system’s health maintenance organization, for clinical and cost data accessibility • exclusion: history of severe neurologic diseases • mean age 56.9; 67.2% female	resource use for imaging in rate of population and monetised cost aggregated to a clinic and projected to positive yield
Ammar, H., et al.,2017 [34]; USA	retrospective chart review	emergency department	January 1, 2011 – December 31, 2011	ICD-9 codes 780.4 (Dizziness and giddiness), 781.2 (Abnormality of gait, 386.0 (MD), 386.1 (Other and unspecified peripheral vertigo), 386.2 (Vertigo of central origin)	521 patients • inclusion: ED visit due to dizziness; age ≥ 18 • exclusion: syncope • mean age 49.3; 57.8% female	• resource use for specialist visit in rate of population • resource use for imaging in rate of population and monetised cost aggregated to a clinic and projected to positive yield • resource use for hospitalisations in number of occasions per patient and in rate of population
Benecke, H., et al., 2013 [35]; Czech Republic, Germany, Hungary, Slovenia	multi-country, observational, data of Registry to Evaluate the Burden of Disease in Vertigo	general practitioners, specialists (ear-nose-throat, neurologist), emergency department	April 20, 2007 - August 15, 2009	MD, BPPV, other vertigo of peripheral vestibular origin, or peripheral vestibular vertigo of unknown origin	4294 patients of incident vertigo included in the registry, of which from • Czech Republic 559 • Germany 99 • Hungary 1320 • Slovenia 130 • 65.3% female	• resource use for primary care, specialist, and ED visits and hospitalisations in number of occasions per patient • indirect: work absenteeism in number of occasions per patient; disability in rate of population
Gandolfi, M.M., et al.,2015 [36]; USA	retrospective chart review	academic specialty centre	January 1, 2010 - August 30, 2013	Unilateral Vestibular Weakness	• 1358 included patients from 1996 vertigo patients • inclusion: visit due to vertigo, unilateral caloric weakness (≥20%), abnormal ocular motor testing, and nystagmus on positional testing • mean age 62; 69.4% female	resource use for imaging in rate of population and monetised cost aggregated to a clinic and projected to positive yield
Grill, E., et al.,2014 [37]; Germany	retrospective cohort study; patient-reported questionnaire data	all level health services utilization prior to visiting a tertiary care centre	2011 to 2012	BPPV, MD, vestibular paroxysmia, functional vertigo, bilateral vestibulopathy, vestibular migraine	2374 patients of a tertiary vertigo centre as convenience sample • inclusion: age ≥ 18 • mean age 55.3; 59.8% female	resource use for primary care visits, diagnostic investigations, imaging, medication, and therapeutic measures in rate of population
Lin and Bhattacharyya, 2011 [38]; USA	retrospective chart review on national level from National Ambulatory Medical Care Survey	all outpatient visits, national level	2005 to 2007	ICD–9 codes 386.00, 386.03 (MD), 386.12 (Vestibular neuritis), 386.11 (BPPV), 386.10, 386.19, 386.20, 438.85, 386.10 (other vertigo); [sensorineural hearing Loss]	4.48 million outpatient visits due to an otologic diagnosis; patient number not provided • age ≥ 65 • mean age 77.4, 63% female	resource use in rate of population for all outpatient and specialist visits, and in number of occasions aggregated to national level
McDowell, T. and F. Moore, 2016 [39]; Canada	retrospective chart review	emergency department	1 January 1, 2011 - December 31, 2011	ICD–9 codes 386.11 (BPPV) or 780.4 (Dizziness and giddiness)	642 included patients from 1196 vertigo patients • exclusion: non-neurovestibular vertigo • mean age 63; 60.3% female	resource use for imaging, specialist visit, and diagnostic investigation in rate of population
Neuhauser, H.K., et al.,2008 [40]; Germany	cross-sectional, questionnaire data from the National Health Interview Survey	German representative sample	12 months prior to the interview in 2003	MD, migrainous vertigo, orthostatic dizziness, and BPPV	1003 individuals with dizziness/vertigo from the 4869 participants • 243 vestibular vertigo • 742 nonvestibular dizziness • 18 uncatogorised • age range 18–79 years	• resource use for primary care and specialist visits, and hospitalisation in rate of population • indirect: work absenteeism and disability in rate of population
Reddy et al., 2011 [41]; UK	prospective cohort	nurse-led dizziness clinic	July 2007 to May 2009	BPPV	99 consecutive patients • 25 males, mean age 61 years; • 74 females, mean age 58.2	monetised cost per patient for specialist visit
Saber Tehrani, A.S., et al., 2013 [42]; USA	time-series cost analysis; prevalence data from the National Hospital Ambulatory Medical Care Survey (1995–2000, 2005–2009); cost data (2003 to 2008) from the Medical Expenditure Panel	emergency department, national level	estimated for 2011	ICD-9 codes 780.4 (Dizziness and giddiness) or 386.x (Vertiginous syndromes and other disorders of vestibular system)	12,202 dizziness visit and 360,424 non- dizziness visits in 15-years • inclusion: age ≥ 16	• resource use in number of occasions and in rate of visits for ED visit aggregated to national level • monetised cost per patient and aggregated to national level for ED visit • resource use in rate of population and in monetised cost aggregated to national level for imaging
Skoien et al., 2008 [43]; Norway	register-based prospective study, National Insurance Services data	national level	1996–2002	H82 (vertiginous syndrome), and N17 (vertigo/dizziness)	694 female and 326 male vertigo patients from 920,139 women and 1,019,216 men	indirect: disability in rate of population
Sun, D.Q., et al., 2014 [44]; USA	cross-sectional; patient-reported Dizziness Handicap Index and Health Utilities Index Mark 3; costs based on Medicare reimbursement figures and US Bureau of Labor Statistics data	academic medical centre	12 months prior to the survey in 2013	Vestibular deficiency including MD, verified by caloric nystagmography	15 patients with bilateral vestibular deficiency (BVD) and 22 patients with unilateral vestibular deficiency (UVD); 23 healthy controls • BVD: mean age 65, 27% female UVD: mean age 62, 59% female	• resource use in number of occasions and monetised cost per patient for ED visit, and hospitalisation • Indirect: work absenteeism and consequences in number of occasions per patient
Tyrrell et al., 2016 [45]; UK	retrospective chart review, data from UK Biobank 2007–2012; Hospital Episode Statistics, UK Meniere’s Society.	national estimation	2013–2014	ICD-10 H810 (MD)	1376 patients from 502,682 UK Biobank participants • 37–73 years • Extrapolated toyearly incidence: 2719 cases (i.e. 4.3 per 100,000 estimated prevalence: (0.25%; ~ 162,000 patients)	• resource use in number of occasions per patient and monetised costs aggregated to national level for primary care, specialist, and ED visits • monetised costs aggregated to national level for imaging, diagnostic investigation, medication, therapeutic measures, and total direct cost • Indirect: work absenteeism, disability, and consequences in monetised costs aggregated to national level
Wiltink, J., et al., 2009 [46]; Germany	cross-sectional interview-based survey	German representative sample	12 months prior to the survey in 2006	patient-reported dizziness in the past 4 weeks	201 dizziness patients from 1269 participants • all participants: mean age 48.8; 54.6% female • vertigo patients: mean age 58.8; 59.7% female	• resource use in number of occasions per patient and in rate of population for primary care visits • resource use in rate of population for hospitalisation, medication, and therapeutic measures • Indirect: consequences in rate of population
Yardley et al., 2012 [47]; UK	three arm, pragmatic, randomised controlled trial	primary care	October 2008 to January 2011	Vestibular vertigo, assessed by the primary health care provider	337 patients participating in any of the survey waves; 263 in all • inclusion: vertigo-related diagnostic and medication terms in practice records • exclusion: non-labyrinthine cause of dizziness, serious comorbidity, language barriers • mean age 59.4; 71% female	monetised total direct cost per patient

*BPPV* Benign Paroxysmal Positional Vertigo, *BVD* bilateral vestibular deficiency, *UVD* unilateral vestibular deficiency, *MD* Meniere’s disease, *ED* emergency department

Regarding the type of the vertigo, addressed diseases and symptoms ranged from unspecified patient-reported dizziness [46] to verified vestibular diseases such as uni- or bilateral vestibular deficiency (UVD, BVD) [36, 44], MD [45], and BPPV [41]. Most of the studies covered a range of central and peripherial vestibular vertigo [40], including undiagnosed vertigo [32–35, 38, 39, 42, 43, 47]; and including functional vertigo as well [37].

The studies reported the burden of vertigo by variuos means: resource use per patient, aggregated to or in rate of a certain population; monetised cost either measured or estimated per patient, per diagnostic investigation and/or per positive yield, or on different agggregation level. The applied population covered a wide range from general population, nationally representative patient sample or overall national health care data, or patient population diverse in terms of the above detailed diagnoses and health care services (Table 1).

With two exceptions [41, 47], the studies were observational; quality criteria items referring to an intervention being not applicable. This resulted in a median quality score of six from 19 total of the CHEC (Additional file 2). One of the studies [44] conducted a sensitivity analysis; one study [42] applied inflation adjustment.

### Direct costs of vertigo

Two studies calculated the overall annual cost per patient [44, 45], and seven studies reported specific monetised cost components [33, 34, 36, 41, 42, 44, 47]. Seven studies reported health care utilization [32, 35, 37, 39, 40, 43, 46]. Table 2 demonstrates the detailed direct costs (both in original currency and in 2016 USD) and/or health care utilization data reported by the studies.

**Table 2:** Direct costs of vertigo

Type of health service	Resource use	In % for reported population	Costs [converted to 2016 USD^a^]
Medical consultations			
Per person			
Primary care provider	Within 3 months: 1.1 (Czech Republic), 1.8 (Germany), 2.6 (Hungary), 2.4 (Slovenia) (Benecke et al., 2013 [35]) Per year: 5 (Tyrrell et al., 2016 [45]), 6.6 (with comorbid anxiety), 6.4 (without comorbid anxiety) (Wiltink et al., 2009 [46])		
Specialist	Within 3 months: 1.8 (Czech Republic), 1.2 (Germany), 1.2 (Hungary), 0.8 (Slovenia) (Benecke et al., 2013 [35]) Per year: 1 (Tyrrell et al., 2016 [45])		76 [123] GBP (follow-up visit) (Reddy et al., 2011 [41])
Emergency department	Within 3 months: 0.3 (Czech Republic), 0.2 (Germany), 0.4 (Hungary), 0.6 (Slovenia) (Benecke et al., 2013 [35]) Per year 0.3 (0–3) (BVD), 0.1 (0–2.4) (UVD) (Sun et al., 2014 [44])		Per year: 274 [289] USD (BVD), 94 [99] USD (UVD) (Sun et al., 2014 [44]), 1004 [1077] USD (any dizziness), 768 [824] USD (otologic / vestibular cause) (Saber Tehrani et al., 2013 [42])
Hospitalisation	Within 3 months: 1.7 (days, Czech Republic), 0.4 (days, Germany), 1.0 (days, Hungary), 0.8 (days, Slovenia) (Benecke et al., 2013 [35]) Per year: 2.7 (days, all ED vertigo), 6.7 (days, central neurological vertigo), 2.3 (days, non-central vertigo) (Ammar et al., 2017 [34]) Per year: 1.4 (occasions, BVD), 0.7 (occasions, UVD) (Sun et al., 2014 [44])		Per year: 203 [214] USD (BVD), 92 [97] USD (UVD) (Sun et al., 2014 [44])
Aggregated			
Primary care provider		14.3% (all outpatient visits) (Lin and Bhattacharyya, 2011 [38]), 1.8% (incident vertigo, general population), 17.1% (lifetime) (Neuhauser et al., 2008 [40]), 61.3% (> 2 visits) (Grill et al., 2014 [37]), 57.1% (with comorbid anxiety), 33.1% (without comorbid anxiety) (Wiltink et al., 2009 [46])	Per year: 35.54 [51.75] million GBP (Tyrrell et al., 2016 [45]
Specialist		4.2% (neurology), 1.3% (ENT) (Ammar et al. 2017 [34]), 16.4% (neurology) (McDowell and Moore, 2016 [39]) 30% (neurology, lifetime, vestibular vertigo), 12% (neurology, lifetime, non-vestibular vertigo), 34% (ear-nose-throat, lifetime, vestibular vertigo), 7% (ENT, non-vestibular vertigo) (Neuhauser et al., 2008 [40]) 57.0% (otolaryngology), 21.0% (internal medicine), 2.2% (neurology), 1.2% (cardiovascular) (Lin and Bhattacharyya, 2011 [38])	Per year: 10.0 [14.56] million GBP (Tyrrell et al., 2016 [45])
Emergency department	Per year: 3.9 million (Saber Tehrani et al., 2013 [42])	25.7% (all ED visits), trend from 2.7% in 1995 to 3.8% in 2015 (costs, all ED visit) (Saber Tehrani et al., 2013 [42])	Per year: 3.9 [4.2] billion USD (Saber Tehrani et al., 2013 [42]), 0.68 [0.99] million GBP (Tyrrell et al., 2016 [45])
Hospitalisation		24.6% (ED vertigo) (Ammar et al., 2017 [34]), 10% (lifetime, vestibular vertigo), 5% (lifetime, non-vestibular vertigo) (Neuhauser et al., 2008 [40]), 8.9% (with comorbid anxiety), 2.8% (without comorbid anxiety) (Wiltink et al., 2009 [46])	
All visits	Per year: 292,077 (MD), 262,878 (vestibular neuritis), 230,311 (BPPV), 10,143 (vertigo), 1.218 million (all, forecasted by 2020) (Lin and Bhattacharyya, 2011 [38])		
Diagnostic investigations			
Per person			
CT			1220 [1265] USD, 164,700 [176,720] USD (positive yield) (Ahsan et al., 2013 [33]), 50,830 [54,540] USD (positive yield) (Ammar et al., 2017 [34])
MRI			2696 [2795] USD, 22,058 [23,668] USD (positive yield) (Ahsan et al., 2013 [33]), 33,575 [36,025] USD (positive yield) (Ammar et al., 2017 [34]), 15,180 [15,737] USD (positive yield) (Gandolfi et al., 2015 [36])
All neuroimaging			39.976 [41,442] USD (positive yield) (Ahsan et al., 2013 [33])
Other	1 (audiology) (Tyrrell et al., 2016 [45])		
All investigations	3.2 (instrumental diagnostic procedures) (Grill et al., 2014 [37])		
Aggregated			
HIT		5% (McDowell and Moore, 2016 [39])	
CT		48% (Ahsan et al., 2013 [33]), 42% (Ammar et al., 2017 [34]), 31% (episodic vertigo), 50.8% (acute constant vertigo), 60.9% (chronic vertigo) (McDowell and Moore, 2016 [39])	Per year: 360 [386] million USD (Saber Tehrani et al., 2013 [42]), 406,646 [436,324] USD (Ammar et al., 2017 [34]) Per 3 years: 988,200 [1,060,322] USD (Ahsan et al., 2013 [33])
MRI		9.5%, (Ammar et al., 2017 [34]), 5.3% (Ahsan et al., 2013 [33]), 18.6% (Gandolfi et al., 2015 [36]), 1.2% (episodic vertigo), 9% (acute constant vertigo) (McDowell and Moore, 2016 [39]), 76.2% (Grill et al., 2014 [37])	Per year: 201,450 [216,153] USD (ED) (Ammar et al., 2017 [34]), 110 [118] million USD (Saber Tehrani et al., 2013 [42]), 0.38 [0.55] million GBP (MD, incident cases) (Tyrrell et al., 2016 [45]) Per 3 years: 242,640 [260,349] USD (ED) (Ahsan et al., 2013 [33]), 303,600 [333,147] USD (ED) (Gandolfi et al., 2015 [36])
All neuroimaging		12% (total costs, ED visits), trend from 10.0% in 1995 to 47.9% in 2015 (ED vertigo) (Saber Tehrani et al., 2013 [42]), 82% (tertiary vertigo centre patients) (Grill et al., 2014 [37])	Per 3 years: 1,230,840 [1,275,985] USD (ED), ~ 1,2 [1.24] million USD (potential savings on unremarkable imaging) (Ahsan et al., 2013 [33])
other		2.30% (basic vestibular evaluation), 1.96% (caloric test), 1.06% (rotary chair test) (Adams et al., 2017 [32]), 59% (complete neurological examination) (Ammar et al., 2017 [34]), 53.5% (electrocardiography) (Grill et al., 2014 [37]), 31.4% (Dix-Hallpike manoeuvre) (McDowell and Moore, 2016 [39])	Per year: 0.15 [0.22] million GBP (hearing test, incident cases), 0.61 [0.89] million GBP (audiology) (Tyrrell et al., 2016 [45])
Therapy			
Per person			
Medication	1.8 (number of medicines) (Grill et al., 2014 [37])		
Aggregated			
Medication		61.0% (all), 25.9% (betahistine), 37.3% (homeopathic) (Grill et al., 2014 [37]), 0.4% - 45% (prevention of attacks) (Tyrrell et al., 2016 [45]), 44.6% (psychiatric, with comorbid anxiety), 12.1% (psychiatric, without comorbid anxiety), 26.8% (dizziness, with comorbid anxiety), 13.5% (dizziness, without comorbid anxiety) (Wiltink et al., 2009 [46])	Per year: 7.90 [11.72] million GBP (all), 4.19 [6.21] million GBP (betahistine), 1.76 [2.61] million GBP (prochlorperazine), 0.22 [0.33] million GBP (bendrofluazide), 1.63 [2.42] million GBP (cinnarizine), 0.05 [0.07] million GBP (buccastem), (0.06 [0.09] million GBP (cyclizine) (Tyrrell et al., 2016 [45])
Other		15.3% (Epley manoeuvre, BPPV) (McDowell and Moore, 2016 [39]), 21.4% (psychotherapy with comorbid anxiety), 5.7% (psychotherapy without comorbid anxiety) (Wiltink et al., 2009 [46]), 41.3% (physical therapy) (Grill et al., 2014 [37])	Per year: 3.1 [4.6] million GBP (hearing aids) (Tyrrell et al., 2016 [45])
Total direct cost			
Per person			Per year: 35 [53.79] GBP (routine care) (Yardley et al., 2012 [47])
Aggregated			Per year: 61.3 [89.26] million GBP (Tyrrell et al., 2016 [45])

*BVD* bilateral vestibular deficiency, *BPPV* Benign Paroxysmal Positional Vertigo, *CT* Computed Tomography, *ED* Emergency department, *ENT* ear-nose-throat, *GBP* Great Britain pound, *HIT* head impulse test, *MRI* Magnetic Resonance Imaging, *MD* Meniere’s disease, *USD* United States dollar; *UVD* unilateral vestibular deficiency

^a^CCEMG – EPPI-Centre Cost Converter (Shemilt et al. 2010 [30])

#### Medical consultations

From the unselected adult (17–79 year-old) population 1.8% had a consultation in the previous 12 months [40]. In Germany these consultations were roughly equally distributed between primary health care and specialists [40]; in the USA the contribution of primary health care providers was 14.3% [38].

Separated according to the level of health care, the range of primary care consultations was 1.1–2.6 occasion, and the range of specialist consultation was 0.8–1.8 [35] within the 3 month observation period in European countries. More than two consultations occurred in 61.3% of the vertigo patients [37]. Vestibular vertigo patients had more consultations than patients with non-vestibular vertigo [40]. Co-morbid anxiety increased the number of consultations up to 6.6 (SD 5.4) within the previous 12 months [46]. Higher age also contributed to more consultations [35, 40].

#### Emergency care

Occasions of emergency care visits ranged from 0.1 to 0.6 [35] within 3 months in European countries, or 0.1 (SD 0.5) to 0.4 (SD 0.8) within 12 months [44] in the USA, the latter corresponding to mean costs of 94–274 (range 0–2374) USD per case [44]. In a USA national overview, 3.9 million emergency care visits in 2011 resulted in 3.9 billion USD total costs, i.e. on average 1004 USD per patient and visit [42].

#### Hospitalisation

Hospitalisation in Germany due to vertigo was reported by 1.9% of the unselected adult population [40]. Lifetime hospitalisation occurred in 10% of vestibular vertigo patients and in 5% of patients with non-vestibular vertigo [40].

The number of hospital days ranged from 0.4 to 1.7 days during a 3 month observation period [35] in European countries. In the USA, BVD patients had 1.4 (SD 0.8) hospital visits within 12 months, causing a 203 (range 0–348) USD cost [44]. Advanced age contributed to longer hospital stay [35].

#### Diagnostic procedures

Patients in Germany underwent on average 3.2 (range 0–6) instrumental diagnostic procedures [37], the older age group receiving significantly less [32, 37]. Economic reporting about diagnostic procedures focused on imaging, being the most frequently used [37], and the most expensive diagnostic procedures accounted for 12% of the total annual costs for dizziness visits [42]. Prior to referral to a German tertiary balance centre, 82% of the patients received either magnetic resonance imaging (MRI) or computed tomography (CT) [37].

CT was performed in 22.8% [42] and up to 50% [39] of the vertigo patients, depending on the setting, i.e. general patient population or emergency patients, respectively. Each 10-years step in age increased the probability of scanning to 1.4 times [33]. CT use showed an increasing trend in a national-level USA study [42], with an almost two-thirds increase between 2005 and 2009. However, the yielding rate of CT for vestibular diseases was reported to be low, from 0.74% [33] to 3.6% [34]. This resulted in a high diagnostic cost for positive findings, ranging from 54,540 USD [34] to 176,720 USD [33].

MRI was applied in 5.4% [33] to 18.6% [36] of the patients. The yielding rate for vestibular diseases was between 12.2% [33] and 13.8% [36] with a cost range for a positive yield of 15,737 USD [36] to 36,025 USD [34].

Other diagnostic methods were less frequent than imaging: head impulse test was performed in 5% of the patients and the Dix-Hallpike manoeuvre in 31.4% [39]. Among the listed diagnostic methods, head impulse test had the highest yielding rate for vestibular disease of 29% [39].

#### Therapy and medication

Vertigo patients had on average 1.8 (range 0–8) different therapies; the most frequent therapy was medication (61.0% of all patients), with mean of 1.8 (range 0–17) drugs per patient [37]. Physical therapy was prescribed in 41.3% of the patients [37]. The liberatory Epley manoeuvre was performed in 15.3% of BPPV patients [39].

### Indirect costs of vertigo

Loss of working days (Table 3) ranged from 13.1 (SD 14.6) days during a 3 month observation period [35], to 69 (SD 106) days in 12 months [44], the latter corresponding to a mean productivity loss of 12,542 USD [44]. Sick leave was significantly higher in vestibular vertigo patients than in patients with non-vestibular vertigo [40]. Also, 69.8% of the patients reported they had to reduce their workload, 4.6% changed their job and 5.7% quit work [35]. In Norway, almost 1 % of the overall long-term sickness absence was caused by vertigo [43]. Among the affected patients, 23% of women and 24% of men obtained a disability pension [43].

**Table 3:** Indirect costs of vertigo

Type of burden	Resource use	In % for reported population	Costs [converted to 2016 USD^a^]
Work / employment			
Per person	Within 3 months: lost work days 13.1 (Czech Republic), 26.7 (Germany), 13.2 (Hungary), 15.8 (Slovenia) (Benecke et al., 2013 [35]) Per year: lost work days 69 (BVD), 19 (UVD) (Sun et al., 2014 [44])		Per year: 12,542 [13,214] USD (cost of lost work days, BVD), 3345 [3524] USD (cost of lost work days, UVD) (Sun et al., 2014 [44])
Aggregated		41% (sick leave, vestibular vertigo), 15% (sick leave, non-vestibular vertigo) (Neuhauser et al., 2008 [40]) (Benecke et al., 2013 [35]) 23% (disability pension, female) and 24% (disability pension, male) (Skoien et al., 2008 [43]) 40% (interruption of daily activities, vestibular vertigo), 12% (interruption of daily activities, non-vestibular vertigo) (Neuhauser et al., 2008 [40])	Per year: 2.87 [4.26] million GBP (disability benefit, MD), 0.56 [0.83] million GBP (additional attendance allowance, MD), 442.70 [656.49] million GBP (loss of earnings, MD-related unemployment) (Tyrrell et al., 2016 [45])
Comorbidity			
Per person	Per year: 19 (falls, BDV), 2 (falls, UVD) (Sun et al. 2014 [44])		
Aggregated		28.3% (comorbid anxiety) (Wiltink et al., 2009 [46])	Per year: 0.32 [0.47] million GBP (depression treatment), 1.91 [2.83] million GBP (depression mortality), 33.9 [50.3] million GBP (pain and suffering, median willingness to pay), 101.48 [150.49] million GBP (pain and suffering, mean willingness to pay) (Tyrrell et al., 2016 [45])

*BVD* bilateral vestibular deficiency, *GBP* Great Britain pound, *MD* Meniere’s disease, *USD* United States dollar, *UVD* unilateral vestibular deficiency

^a^CCEMG – EPPI-Centre Cost Converter (Shemilt et al. 2010 [30])

Regarding non-monetary burden of disease, both vestibular vertigo patients and patients with non-vestibular vertigo reported avoiding leaving the house (18.5% and 10.1%, respectively), experienced an interruption of daily activities (40.3% and 11.5%, respectively), and perceived reduced quality of life [40] and relevant restrictions in daily life [44]. Concomitant anxiety was reported to further impair social life [46]. Patients with chronic vestibular loss reported an increased frequency of falls [44].

Two studies report on both direct and indirect cost, indicating that the latter may be even higher [44, 45].

### Total costs

Two studies provided a wider view of the total cost of vertigo. A UK study [45] estimated as comprehensive cost of MD 3341–3757 GBP [4865 – 5470] per patients annually. A USA study included emergency visits and hospitalisation plus an estimation of lost working days, adding an average annual economic burden of 13,717 USD per BVD patients [44].

## Discussion

This review is the first to explore the economic burden of vertigo and dizziness including direct and indirect costs. We found large heterogeneity in respect to the investigated sector of the health care system, the type of the vertigo, and the cost components; the quality of studies was mostly mediocre. Annually up to 9.6 visits were reported at the primary care provider [35], up to 7.2 visits at the specialist [35], with up to 6 instrumental diagnostic procedures [37]. Imaging was performed in up to 82% of the patients [37] with a cost of up to 164,700 [176,720] USD per positive yield [33]. Up to 2.4 presentations occurred at the emergency department [35], leading to up to 6.8 hospital days [35]. The trend of the number of ED visits, and imaging due to the vertigo was rising [38, 42]. Studies identified an increase by aging of the population in vertigo prevalence [32, 36, 38], in vertigo-related health care demand [35, 38, 40, 42] and in imaging performed [33]; however, not in the number of other investigations [37].

The original studies reported three main drivers of increased direct costs: unnecessarily repeated consultations of primary, and specialist care, and referrals, where primary care would have been sufficient; excessive use of diagnostic imaging, and excessive use of emergency care, the latter mainly in countries where statutory affordable health insurance is not broadly available.

The number of referrals and consultations largely depends on the respective health care system. Typically, a system of statutory health insurance will regulate reconsultation and referral, however, in rather liberal systems such as Germany, referral is easy and sometimes more cost effective for the primary care physician [48]. Also, in an earlier study we found that primary care providers do not always feel competent to diagnose and treat vertigo [49]. Consequently, patients with vertigo may undergo several consultations and referrals without clear diagnosis or therapy [6, 35, 40].

Second, several studies examined the excess costs of extensive imaging procedures [33, 34, 42]. It is reasonable to assume that physicians do not want to overlook life-threatening diseases. In absence of defined clinical pathways and in systems where imaging is broadly available, imaging seems like an easy solution. In contrast, low-cost examination techniques that require a certain skill set, such as the head impulse test [50] seem to be underutilized [39].

Third, consulting emergency care is not unusual because vertigo is a worrying symptom. However, overuse of the emergency department because of vertiginous symptoms may be a direct result of a system where primary care is not always affordable or available [51].

The limited data on indirect costs due to vertigo indicate that it may be considerable [35, 44, 45].

Considering the three main drivers of direct costs, there is no easy solution to the problem. Original studies recognising the problem of unnecessary or not well-targeted investigations argued for guidelines or managed care [33, 34, 42]. Application of clinical practice guidelines may provide benefit even in terms of preventing unnecessary diagnostic investigations, and supporting evidence-based resource use; thus, contributing to savings [52, 53]. The German Association for Primary Care has put forward a set of guidelines for management of vertigo in the primary care setting [54]. Likewise, guidelines exist in the USA [55], in the Netherlands [56], in Spain [57], in Croatia [58], or in China [59]. Treating vertigo frequently needs a multidisciplinary approach with combined expertise from several medical and therapeutic professions. Capacity building may reduce the vertigo-related direct costs through promoting evidence-based practices and increasing the knowledge base. However, due to the fragmentation of health care, PCPs often lack resources and time to coordinate such a team-based approach [60]. There is therefore an urgent need to focus economic research on the detected fields of main cost drivers, and investigate the effectiveness of care pathways and managed care approaches.

## Limitation

The selected studies targeted various types of vestibular diseases and various cost components, providing a fragmented and incomplete picture. With respect to the limited number of cost reporting, we included studies reporting health care utilisation. Due to difficulties of finding historical unit price information, these were not converted to a monetised form. Though technical and equipment costs might be similar e.g. for imaging, other background aspects of national regulation and financing of the health care system may divert these prices in a broad range [61]. Thus, the financial burden may widely deviate in different countries even by similar prevalence of vertigo and similar frequency of health care utilisation. This limits the value of these data regarding the economic burden of vertigo. The gatekeeping function of primary care and the direct accessibility of specialist consultation is regulated differently depending on the country, thus, the utilisation of consultations on different levels of health care are not directly comparable.

## Conclusion

To the best of our knowledge, this is the first systematic review of the existing evidence of the economic burden of vertigo. Results demonstrated that vertigo contributes to an increasing trend of direct health care costs, due to the ageing of the population. Three main cost drivers due to insufficient diagnostic skills were identified. Repeated and not well-targeted health care consultations on all levels, excessive use of expensive diagnostic imaging, and unnecessary assignment to emergency care. Several studies demonstrated that the ageing of the population contributes to an increasing trend of direct health care costs in persons with vertigo.

The main result, i.e. that there is no conclusive evidence expressing an overall economic burden, may seem disappointing at first. It is, however, the objective of any systematic review not only to summarize existing knowledge but also to summarize apparent gaps of evidence. There are several direct consequences from this gap:

1. Future studies of new or established diagnostic devices and therapies for vestibular disease should include cost-effectiveness considerations.
2. Population-based studies that focus on health care utilization should include simple assessments of vestibular function to get more reliable estimates of burden of disease and associated costs on the level of the general population.
3. Clinical and population-based registries that include patients with vestibular disease, should invest more effort in the exact characterization of disease and consider collecting long-term patient-reported outcomes, absence from work and other types of societal burden.
4. Primary data collection should increasingly include assessment of health care utilization e.g. by linking their diagnoses and outcomes to routine data from health insurances.

## Supplementary information

**Additional file 1:** Detailed search strategy.

**Additional file 2.** Assessment of study quality and risk of bias by the Consensus on Health Economic Criteria*.

## Data Availability

Not applicable.

## Abbreviations

BPPV: benign paroxysmal positional vertigo
BVD: bilateral vestibular deficiency
CCEMG – EPPI-Centre: The Campbell and Cochrane Economics Methods Group and the Evidence for Policy and Practice Information and Coordinating Centre
CHEC: Consensus on Health Economic Criteria
CT: computed tomography
ED: Emergency department
ENT: ear-nose-throat
GBP: Great Britain pound
HIT: head impulse test
ICD: International classification of diseases
MeSH: Medical Subject Headings
MD: Meniere’s disease
MRI: magnetic resonance imaging
UK: United Kingdom
USA: United States of America
USD: United States dollar
UVD: unilateral vestibular deficiency
