# Supplementary material for: Economic burden of vertigo: a systematic review
Source: Health Econ Rev. 2019 Dec 27;9:37. doi: 10.1186/s13561-019-0258-2 (PMC6933936; doi:10.1186/s13561-019-0258-2)
Supplement: Supplementary file 1 — Additional file 1: Detailed search strategy. [file 13561_2019_258_MOESM1_ESM.docx]

## Supplementary material 1. Detailed search strategy

**Search strategy of PubMed database**

((((((((((((((((((BPPV[Title]) OR Meniere[Title]) OR vestib*[Title]) OR labyrinthitis[Title]) OR dizz*[Title]) OR vertigo[Title/Abstract]) OR dizziness[MeSH Terms]) OR vertigo[MeSH Terms]) OR Benign Paroxysmal Positional Vertigo[MeSH Terms]) OR Meniere Disease[MeSH Terms]) OR Vestibular Neuronitis[MeSH Terms]) OR Bilateral Vestibulopathy[MeSH Terms]) NOT schwannoma[Title])) AND (((((((((((((((((((cost[Title/Abstract]) OR financ*[Title]) OR econom*[Title]) OR burden[Title]) OR utilization[Title]) OR pay*[Title]) OR (Fees and Charges[MeSH Terms])) OR Prescription Fees[MeSH Terms]) OR Health Resources/economics[MeSH Terms]) OR Health Resources/utilization[MeSH Terms]) OR Economics[MeSH Terms]) OR Economics, Nursing[MeSH Terms]) OR Economics, Medical[MeSH Terms]) OR Economics, Hospital[MeSH Terms]) OR economics[MeSH Subheading]) OR Vestibular Neuronitis/economics[MeSH Terms]) OR Vertigo/economics[MeSH Terms]) OR Dizziness/economics[MeSH Terms]) OR Meniere Disease/economics[MeSH Terms])) AND ("last 10 years"[PDat] AND English[lang]))

NOT

((systematic review[Title]) OR (“Comment”[Publication Type] OR “Letter”[Publication Type] OR “Editorial”[Publication Type] OR “Clinical Conference”[Publication Type]))

Filters: published in the last 10 years; English

**Search strategy of Cochrane database**

| **No.** | **Search terms in cochrane** | **Results** |
| --- | --- | --- |
| Disease concept | | |
| #1 | "BPPV":ti or "Meniere":ti or vestib*:ti or "labyrinthitis":ti or dizz*:ti (Word variations have been searched) | 1156 |
| #2 | "schwannoma":ti (Word variations have been searched) | 39 |
| #3 | #1 not #2 | 1120 |
| #4 | "vertigo":ti,ab,kw (Word variations have been searched) | 4440 |
| #5 | MeSH descriptor: [Dizziness] explode all trees | 625 |
| #6 | MeSH descriptor: [Vertigo] explode all trees | 418 |
| #7 | MeSH descriptor: [Benign Paroxysmal Positional Vertigo] explode all trees | 43 |
| #8 | MeSH descriptor: [Meniere Disease] explode all trees | 148 |
| #9 | MeSH descriptor: [Vestibular Neuronitis] explode all trees | 17 |
| #10 | MeSH descriptor: [Bilateral Vestibulopathy] explode all trees | 0 |
| #11 | #3 or #2 or #3 or #4 or #5 or #6 or #7 or #8 | 5754 |
| Economic concept | | |
| #12 | MeSH descriptor: [Vertigo] explode all trees and with qualifier(s): [Economics - EC] | 1 |
| #13 | MeSH descriptor: [Dizziness] explode all trees and with qualifier(s): [Economics - EC] | 2 |
| #14 | MeSH descriptor: [Meniere Disease] explode all trees and with qualifier(s): [Economics - EC] | 2 |
| #15 | MeSH descriptor: [Vestibular Neuronitis] explode all trees and with qualifier(s): [Economics - EC] | 1 |
| #16 | MeSH descriptor: [Costs and Cost Analysis] explode all trees | 25708 |
| #17 | MeSH descriptor: [Cost of Illness] explode all trees | 1353 |
| #18 | MeSH descriptor: [Cost-Benefit Analysis] explode all trees | 18591 |
| #19 | MeSH descriptor: [Health Care Costs] explode all trees | 7513 |
| #20 | MeSH descriptor: [Direct Service Costs] explode all trees | 196 |
| #21 | MeSH descriptor: [Hospital Costs] explode all trees | 1532 |
| #22 | MeSH descriptor: [Drug Costs] explode all trees | 1816 |
| #23 | MeSH descriptor: [Health Expenditures] explode all trees | 347 |
| #24 | 16 or 17 or 18 or 19 or 20 or 21 or 22 or 23 | 25708 |
| #25 | MeSH descriptor: [Economics] explode all trees | 27882 |
| #26 | MeSH descriptor: [Economics, Nursing] explode all trees | 21 |
| #27 | MeSH descriptor: [Economics, Medical] explode all trees | 105 |
| #28 | MeSH descriptor: [Economics, Hospital] explode all trees | 1798 |
| #29 | Any MeSH descriptor with qualifier(s): [Economics - EC] | 23839 |
| #30 | 25 or 26 or 27 or 28 or 29 | 31016 |
| #31 | MeSH descriptor: [Health Resources] explode all trees and with qualifier(s): [Economics - EC] | 253 |
| #32 | MeSH descriptor: [Health Resources] explode all trees and with qualifier(s): [Utilization - UT] | 425 |
| #33 | 31 or 32 | 502 |
| #34 | MeSH descriptor: [Fees and Charges] explode all trees | 514 |
| #35 | MeSH descriptor: [Prescription Fees] explode all trees | 24 |
| #36 | 34 or 35 | 514 |
| #37 | cost:ti or financ*:ti or econom*:ti or burden:ti or pay*:ti (Word variations have been searched) | 28231 |
| #38 | "utilization":ti or "utilisation":ti (Word variations have been searched) | 1849 |
| #39 | 35 or 36 | 29842 |
| #40 | 22 or 28 or 31 or 34 or 37 | 41908 |
| #41 | 38 and 9 | 35 |
| #42 | 10 or 11 or 12 or 13 or 39 | 35 |
| Exclusions | | |
| #43 | comment:pt or letter:pt or editorial:pt or clinical conference:pt (Word variations have been searched) | 8907 |
| #44 | "systematic review":ti (Word variations have been searched) | 21736 |
| #45 | #41 or #42 | 30642 |
| Total |  |  |
| #46 | #40 not #43 | 35 |
|  | With filter: Publication Year from 2008 | 15 |

**Search strategy of Embase database**

| 1. ((bppv or Meniere or vestib* or labyrinthitis or dizz* or vertigo).ti. or vertigo.ab.) not schwannoma.ti.  2. *vertigo/  3. *dizziness/  4. *benign paroxysmal positional vertigo/  5. *Meniere disease/  6. *vestibular neuronitis/  7. *bilateral vestibulopathy/  8. 1 or 2 or 3 or 4 or 5 or 6 or 7  9. "health care cost"/ or "cost effectiveness analysis"/ or "hospital cost"/ or "cost minimization analysis"/ or "cost control"/ or "nursing cost"/ or "hospitalization cost"/ or "cost utility analysis"/ or "cost benefit analysis"/ or "cost"/ or "cost of illness"/ or "drug cost"/  10. *health economics/ or *economics/  11. *health care utilization/  12. *health care planning/  13. *medical fee/ or *fee/  14. (cost or financ* or econom* or burden or utilization or utilisation or pay*).ti.  15. 9 or 10 or 11 or 12 or 13 or 14  16. 8 and 15  17. (Comment or Letter or Editorial or Conference).pt.  18. systematic review.ti.  19. 17 or 18  20. 16 not 19  21. limit 20 to (english language and yr="2008 -Current") |
| --- |
