# Supplementary material for: Economic burden of vertigo: a systematic review
Source: Health Econ Rev. 2019 Dec 27;9:37. doi: 10.1186/s13561-019-0258-2 (PMC6933936; doi:10.1186/s13561-019-0258-2)
Supplement: Supplementary file 2 — Additional file 2. Assessment of study quality and risk of bias by the Consensus on Health Economic Criteria*. [file 13561_2019_258_MOESM2_ESM.docx]

## Supplementary material 2 Assessment of study quality and risk of bias by the Consensus on Health Economic Criteria*

|  |  | Adams, M.E., et al., 2017 | Ahsan, S.F., et al. 2013 | Ammar, H., et al., 2017 | Benecke, Agus et al,. 2013 | Gandolfi, M.M., et al.,2015 | Grill, Strupp et al. 2014 | Lin and Bhattacharyya, 2011 | McDowell and Moore 2016 | Neuhauser, Radtke et al. 2008 | Reddy et al., 2011 | Saber Tehrani, Coughlan et al. 2013 | Skoien et al., 2008 | Sun, Ward et al. 2014 | Tyrrell et al., 2016 | Wiltink, Tschan et al. 2009 | Yardley et al., 2012 |
| --- | --- | --- | --- | --- | --- | --- | --- | --- | --- | --- | --- | --- | --- | --- | --- | --- | --- |
| 1 | Is the study population clearly described? | Y | Y | Y | Y | Y | Y | Y | Y | Y | Y | N | Y | Y | Y | Y | Y |
| 2 | Are competing alternatives clearly described? | N.A. | N.A. | N.A. | N.A. | N.A. | N.A. | N.A. | N.A. | N.A. | Y | N.A. | N.A. | N.A. | N.A. | N.A. | Y |
| 3 | Is a well-defined research question posed in answerable form? | Y | Y | Y | Y | Y | Y | Y | Y | Y | Y | Y | Y | Y | Y | Y | Y |
| 4 | Is the economic study design appropriate to the stated objective? | Y | Y | Y | Y | Y | Y | Y | Y | Y | Y | Y | Y | Y | Y | Y | Y |
| 5 | Is the chosen time horizon appropriate to include relevant costs and consequences? | Y | Y | Y | Y | Y | Y | Y | Y | Y | Y | Y | Y | Y | Y | Y | Y |
| 6 | Is the actual perspective chosen appropriate? | N | N | N | N | N | N | N | N | N | N | N | N | N | N | N | N |
| 7 | Are all important and relevant costs for each alternative identified? | N.A. | N.A. | N.A. | N.A. | N.A. | N.A. | N.A. | N.A. | N.A. | Y | N.A. | N.A. | N.A. | N.A. | N.A. | Y. |
| 8 | Are all costs measured appropriately in physical units? | Y | Y | N | N | N | N | Y | N | N | N | Y | N | Y | Y | N | Y |
| 9 | Are costs valued appropriately? | N | N | N | N | N | N | N | N | N | N | Y | N | Y | Y | N | N |
| 10 | Are all important and relevant outcomes for each alternative identified? | N.A. | N.A. | N.A. | N.A. | N.A. | N.A. | N.A. | N.A. | N.A. | N.A. | N.A. | N.A. | Y | N.A. | N | Y |
| 11 | Are all outcomes measured appropriately? | N | N | N | N | N | N | N | N | N | Y | N | Y | Y | Y | N | Y |
| 12 | Are outcomes valued appropriately? | N | N | N | N | N | N | N | N | N | N | N | N | N | Y | N | N |
| 13 | Is an incremental analysis of costs and outcomes of alternatives performed? | N | N | N | N | N | N | N | N | N | N | N | N | Y | N | N | N |
| 14 | Are all future costs and outcomes discounted appropriately? | N | N | N | N | N | N | N | N | N | N | N | N | N | N | N | N |
| 15 | Are all important variables, whose values are uncertain, appropriately subjected to sensitivity analysis? | N | N | N | N | N | N | N | N | N | N | N | N | Y | N | N | N |
| 16 | Do the conclusions follow from the data reported? | Y | Y | Y | Y | Y | Y | Y | Y | Y | Y | Y | Y | Y | Y | Y | Y |
| 17 | Does the study discuss the generalizability of the results to other settings and patient/ client groups? | N | N | N | N | N | N | Y | N | Y | N | N | N | Y | Y | N | Y |
| 18 | Does the article indicate that there is no potential conflict of interest of study researcher(s) and funder(s)? | Y | Y | Y | Y | Y | Y | N | Y | N | Y | Y | Y | Y | Y | N | Y |
| 19 | Are ethical and distributional issues discussed appropriately? | N.A. | N | N | Y | N | Y | N.A. | Y | N | Y | N | N.A. | N | N.A. | N | Y |
|  | total score(*/19) | 7/19 | 7/19 | 6/19 | 7/19 | 6/19 | 7/19 | 7/19 | 7/19 | 6/19 | 10/19 | 7/19 | 7/19 | 13/19 | 11/19 | 5/19 | 12/19 |

*(Evers et al., 2005)

Y yes, N no, N.A. not applicable
